# Massachusetts Veterinary Association

**Published:** 1897-11

**Authors:** H. S. Lewis

**Affiliations:** Secretary


### SOCIETY PROCEEDINGS.

### MASSACHUSETTS VETERINARY ASSOCIATION.

THE monthly meeting was held June 16th at the Quarantine Station, Gallop's Island, which was open to the Association through the courtesy of the Board of Health of the city of Boston. The members left Lincoln Wharf on board of a fine tug, which was placed at their disposal by N. Ward & Co. After an inspection of the different departments, President Winchester called the meeting to order in the laboratory. Members present: Drs. Beckett, Blackwood, Bunker, Burr, Emerson, Frothingham, Hamilton, Howard, Lobour, Lewis, Osgood, Parker, Penniman, Peters, Peterson, Pierce, McLaughlin, Rodgers, Sheldon, Williams, Winchester, Stickney. The Association had as guests: Dr. Monell, of Lawrence; Dr. A. S. Lamb, of Woburn; Mr. Fred. A. Weil, of the Lawrence *American*; and Mr. Andrew Ward.

Dr. Bunker moved that the meeting be considered the regular monthly meeting, and that when we adjourn we adjourn until September.

Dr. Frothingham read a paper on the "Method of Procedure in Preparing Antitoxin," which was very instructive.

Dr. Burr explained the condition of each horse in the stable that is used for serum, and its condition before and after.

The Association then took the tug to Pemberton; from there they took the electric railway to Strawberry Hill, where a shore dinner was served at the Waveland House; after a smoke-talk they were taken in barges through the town of Hull, back to the tug, when a few returned to Boston by the regular boat, and the rest took a moonlight sail out to sea, returning to Boston about 10.30 P.M.

H. S. LEWIS,
Secretary.

